# Do socio-demographic factors predict children’s engagement in arts and culture? Comparisons of in-school and out-of-school participation in the Taking Part Survey

**DOI:** 10.1371/journal.pone.0246936

**Published:** 2021-02-12

**Authors:** Hei Wan Mak, Daisy Fancourt

**Affiliations:** Department of Behavioural Science and Health, Institute of Epidemiology & Health Care, University College London, London, United Kingdom; MacEwan University, CANADA

## Abstract

There is evidence on the health, social and developmental benefits of arts and cultural participation for young people. While there is a known social gradient across adult arts participation where socially advantaged individuals are more likely to engage in the arts, it remains unclear whether socio-economic factors also affect child participation either in school or out of school. This study analysed cross-sectional data from 1,986 children aged 11–15 in the Taking Part Survey interviewed from 2015–2018. It focused on three aspects of children‘s participation: (i) performing arts activities, (i) arts, crafts and design activities, and (iii) cultural and heritage engagement. Results show a social gradient across all three activities for out-of-school engagement, but not for in-school engagement. Arts and cultural activities provided by schools are therefore important to ensuring universal access to the arts amongst young people.

## Introduction

Adolescence is a transitional period from childhood to adulthood in which young people experience biopsychosocial development, influencing their cognitive ability, efficiency of thought, reasoning, emotional lability, autonomy, self-identity and social identity [1]. There is a wide literature on the benefits arts and cultural participation that help support adolescent development. Arts and cultural participation consists of different activities including *performing arts* (e.g. dancing, singing), *visual and literary arts* (e.g. textiles, painting, writing stories), and *culture* (e.g. going to museums, galleries, the theatre, heritage sites) [2]. Previous literature has shown that musical activities [3–5], dancing [6], museum engagement [7], and cultural activities [8] help improve reading ability, academic performance across school years, and raise educational aspiration. Visiting museums and archives help youth develop critical thinking, engage in meaningful learning activities, facilitate positive relationships with peers and allow them to be exposed to positive values [9, 10]. Further, it has been shown that arts engagement (including musical activities, dance, drama, reading for pleasure, visual arts and museum and archives engagement) can improve classroom behaviours such as attention [11, 12], as well as promoting prosocial behaviours [13–16], enhancing emotional competency [17, 18], reducing competitive dynamics in classrooms [19], and reducing bullying [20–23]; all of which support educational outcomes. It has also been demonstrated that art classes could help support students’ creative interests and self-reflection and help cultivate their artistic interests that may have implications on other subjects [24]. Beyond learning, the arts have also been found to improve physical and mental health in young people. Previous studies show that performing arts engagement and reading for pleasure may help support physical health, with associations with increased fruit consumption, reduced cigarette and alcohol use, improved cardiovascular fitness and body composition, and reduced body mass [25–27]. Evidence has also demonstrated that engagement with the arts is associated with reduced social and behavioural maladjustment [28], higher resilience and self-esteem [27, 29–31], and lower levels of anxiety and depression [32–34]. While much of this research has come from observational studies, these studies are increasingly showing associations in longitudinal data and independent of identified confounders.

However, despite the wide range of potential benefits of the arts for young people, it is likely that there is a social gradient across arts participation as has also been shown in adult population. Previous research has shown that women and individuals of higher socio-economic status (SES) and with higher levels of academic attainment are more likely to engage, while individuals who are older and from ethnic minority groups are less likely to engage [2, 35–37]. Yet little research has been carried out to ascertain whether similar factors affect participation amongst children [35, 37, 38]. Further, amongst children, there are different arenas for engaging in arts activities, both out of school and in school. Out of school engagement is when children undertake activities outside of the formal and compulsory classes (e.g. extracurricular activities). It has been suggested that this type of engagement tends to follow a similar pattern and have similar predictors as the engagement amongst the adult population. For example, a national study published by the UK Social Mobility Commission found that children from the highest income households are nearly 3 times more likely to participate in music activities and twice as likely to engage in dancing outside school, respectively, compared to the lowest income households [5]. Girls are also more likely to take part in art, music, and dancing activities than boys. Similarly, engagement rates in art and dance are also higher amongst youth from a white ethnic background [5].

However, in contrast to out-of-school engagement, patterns of in-school engagement may be more ubiquitous. In the UK, Arts and Design and Music programmes are provided in the national curriculum to pupils in key stages 1 to 3 (ages between 5 and 14); participation is usually mandatory. In school, children may have more equal access to arts materials (such as paints, clays, sketch books, musical instruments etc.) and be actively encouraged to engage in lessons. In addition, while educational school trips (e.g. visiting museums, galleries, historical sites) are not included in the national curriculum, most schools offer such experiences, often with subsidies, bursaries or free places to make the trips more affordable to many pupils. However, in key stage 4 (aged 14–16), such opportunities may become more sporadic as schools and pupils can select whether or not they take arts-based subjects.

Consequently, whilst it is predicted that the same social factors that affect adult engagement with the arts reproduce unequal engagement amongst children outside of school [39], less is known about whether the same inequities in engagement persist in academic settings. Understanding what influences children’s arts engagement in different contexts is important given that childhood experiences and skills could have a durable impact on their later life outcomes. Therefore, in this paper, we used a national sample of young people aged 11–15 living in the UK to explore the frequency of arts participation and cultural engagement both in school and out of school and to identify whether socio-demographic factors predict engagement.

## Materials and methods

This study analysed data from the Taking Part, a nationally representative survey commissioned by the UK Department for Digital, Culture, Media and Sport (DCMS), in partnership with Arts Council England, Historic England and Sport England. Taking Part covers a wide range of topics in relation to leisure, cultural and sporting participation in England, including arts participation, and cultural and heritage engagement. The survey started with approximately 28,000 adults in England in 2005 and collects data annually. From 2006, Taking Part has initiated a children questionnaire by randomly selecting children aged 11–15 in surveyed households[40–45]. This study focused on nationally-representative cross-sectional samples interviewed in the 2015/16, 2016/17 and 2017/18 surveys. A total of 10,041 adults (aged 16+), 9,277 adults and 7,715 adults participated in the 2015/16, 2016/17 and 2017/18 surveys, respectively. In this study, we only considered participants with children who were aged 11–15 and had completed the children questionnaires. This provided 680 participants in the 2015/16 survey, 635 in the 2016/17 survey, and 671 in the 2017/18 survey. We combined the data from these three waves, providing a total sample of 1,986 children.

The Taking Part survey has been approved by NatCen’s Social Research Ethics Committee. Consent for the interview was obtained verbally and parental permission was recorded at the appropriate point in the instruments for participants under age 16.

### Measurements

This study focused on three main aspects of children arts and cultural participation: (i) engaging in performing arts activities (including dance activities e.g. taken part in a dance club or in a dance performance, music activities e.g. sang to an audience or rehearsed for a performance, and theatre and drama activities e.g. taken part in drama lesson or attended theatre performances such as plays, pantomime, opera, musicals or comedy), (ii) engaging in arts, crafts and design activities (such as painting, drawing, sculpture or model making or attended exhibition of arts, photography or other craft work), and (iii) visiting an archive, a museum or a heritage site (including a historic building, garden or landscape open to the public or a city or town with historic character). For each aspect, children respondents were asked whether they had participated in any of the activities in the past 12 months. A full detail on the activities of these three sets of children’s arts and cultural participation can be found in Appendix A. Respondents were then asked how often they had done the activity/activities in the past 12 months on a four-point scale ranging from ‘at least once a week’ to ‘ 1–2 times a year’. This set of questions was asked twice: once for activities carried out ‘in school’ (i.e. school lessons) and once for activities carried out ‘outside of school’. We categorised responses into binary categories indicating ‘frequent’ vs ‘infrequent’ engagement (arts participation: at least once a week vs less often; culture and heritage engagement: at least 3–4 times a year vs less often).

To understand patterns of variation in-school and out-of-school engagement, we considered a set of socio-demographic predictors. These included children’s gender and ethnicity (white ethnic vs ethnic minority), parents’ marital status (married or in cohabitation vs not married), parental socio-economic status (SES) (assessed using the National Statistics Socio-economic Classification (NS-SEC); high managerial, administrative and professional occupations vs intermediate occupations vs routine and manual occupations or never worked or long-term unemployed), parents’ employment status (working full-time or part time vs not in employment (including students or retired)), parents’ educational level (degree vs no degree), levels of area deprivation (a 3-point scale, i.e. 30% most deprived vs medium vs 30% least deprived, which was derived from the original 10-point scale that is comprised of 7 domains of deprivation, e.g. living environment, income, employment and crime), tenure (private rented sector or housing owner vs social rented sector, i.e. accommodation that is owned and managed by either local authorities or housing associations) and living area (urban vs rural).

Given that there may be intergenerational transmission of arts and cultural engagement, we additionally considered two set of variables on parents’ engagement in these activities. First, we measured parents’ arts engagement when they were growing up (aged 11–15), including participation in performing arts activities (e.g. going to theatre or to see dance or classical musical performances, and playing musical instrument(s), acting, dancing or singing); engagement in arts and crafts (drawing or painting), and engagement in visiting museums or art galleries or heritage sites (such as historic attractions e.g. old buildings, historic parks and gardens and archaeological sites). Second, we assessed parents’ engagement in each of these activities voluntarily or during their own time in the past 12 months. A full description of parental previous and current engagement in the arts can be found in Appendix B.

### Analysis

To examine the associations between socio-demographic factors and arts and cultural engagement during school time and free time, we used multivariable logistic regression analyses. A total of 611 individuals were missing data on one or more variables (30.8%). So multiple imputation by chained equations using the predictor variables outlined above were used to create 50 imputed datasets to account for the missingness. Imputed and non-imputed data provided similar results, so we report findings from the imputed data set. All analyses were weighted using the weights supplied by the survey administrators to ensure our sample was representative [40]. Odds ratios (OR) are presented in our models, showing the odds that a young person would be frequently engaged in the arts depending on each socio-demographic factor. Given that arts and cultural engagement at school and outside school is likely to be related at the area level, the 95%CI in regression models were calculated by clustering standard errors within area and year using the area code provided by the data set that also accounts for the year of the survey fieldwork.

In our analyses, model 1 included socio-demographic characteristics, while model 2 included parental arts engagement whilst adjusting for socio-demographic characteristics. All analyses met regression assumptions and were carried out using Stata v15.

## Results

In our sample, 50% were female, 73% were White, 78% of the children had parents who were married or in cohabitation and 45% had parents with higher managerial, administrative and professional occupations. 61% had participated in performing arts activities with school at least once a week and 31% participated during their own time. Similarly, 56% of the children had engaged in arts, crafts and design activities at school frequently and 19% had engaged during their own time. 13% had visited archives, museums or heritage sites with school and 36% had visited these places during their own time. (Table 1). The distribution of arts and cultural engagement during school time and free time by socio-demographic factors are shown in S1–S3 Tables.

**Table 1:** Descriptive statistics of children’s engagement in arts and cultural activities in and out of school (aged 11–15) (%).

	In school (N = 1986)		Out of school (N = 1986)	
	Infrequently	Frequently	Infrequently	Frequently
Performing arts activities (at least once a week vs otherwise)	39.4	60.6	69.0	31.0
Arts, crafts and design activities (at least once a week vs otherwise)	44.4	55.6	80.6	19.4
Visiting an archive, a museum or a heritage site (at least 3–4 times a year vs otherwise)	87.2	12.8	63.7	36.3

### Out-of-school engagement

Outside of school, there was evidence of a social gradient across all three activities. Girls were more likely than boys to engage in all activities, with 3.4 times higher odds of engaging in performing arts activities (OR = 3.43, CI = 2.51–4.68), 2.9 times higher odds for arts, crafts and design activities (OR = 2.90, CI = 2.08–4.03) and 1.5 times higher odds for archive, museum or heritage engagement (OR = 1.45, CI = 1.11–1.91). Children from an ethnic minority background had a 48% lower odds of visiting an archive, a museum or heritage site, compared to those from a white majority background (OR = 0.52, CI = 0.35–0.79) (Tables 2–4).

**Table 2:** Logistic regressions estimating the association between socio-demographic backgrounds and children’s engagement in performing arts activities in and out of school once a week in the past 12 months.

	In school (at least once a week vs otherwise)			Out of school (at least once a week vs otherwise)		
	OR	95%CI	P-value	OR	95%CI	P-value
***Model 1***						
*Sex*						
Male	REF			REF		
Female	**1.79**	**1.36–2.37**	**0.000**	**3.43**	**2.51–4.68**	**0.000**
*Ethnicity*						
White ethnic	REF			REF		
Ethnic minority	0.89	0.61–1.30	0.557	0.75	0.49–1.13	0.169
*Parents’ marital status*						
Married/in cohabitation	REF			REF		
Single and never married or separated or divorced or widowed	1.07	0.78–1.46	0.692	0.96	0.65–1.44	0.857
*Socio-economic status*						
Higher managerial, administrative and professional occupations	REF			REF		
Intermediate occupations	1.01	0.69–1.47	0.974	0.74	0.48–1.15	0.185
Routine and manual occupations or never worked or long-term unemployed	0.77	0.53–1.13	0.179	**0.60**	**0.38–0.93**	**0.022**
*Parents’ working status*						
Working full-time/part-time	REF			REF		
Not in employment (including students/retired)	0.98	0.64–1.49	0.911	0.94	0.58–1.53	0.800
*Parents’ educational level*						
Degree	REF			REF		
No degree	0.84	0.60–1.18	0.314	0.71	0.49–1.02	0.067
*Levels of area deprivation*						
30% most deprived	1.08	0.76–1.54	0.677	0.91	0.59–1.41	0.689
Medium	REF			REF		
30% least deprived	1.23	0.89–1.70	0.202	**2.09**	**1.42–3.07**	**0.000**
*Tenure*						
Private rented sector or house owning	REF			REF		
Social rented sector	0.96	0.66–1.38	0.817	0.62	0.36–1.08	0.090
*Living area*						
Urban	REF			REF		
Rural	0.82	0.57–1.20	0.314	1.17	0.76–1.79	0.475
***Model 2***						
*+ Parents have done performing arts while growing up*	1.21	0.88–1.65	0.243	1.42	0.95–2.11	0.089
*+ Parents have done performing arts in the past 12 months*	1.31	0.96–1.79	0.086	**2.22**	**1.46–3.37**	**0.000**
**N**	**1986**			**1986**		

**Table 3:** Logistic regressions estimating the association between socio-demographic backgrounds and children’s engagement in arts, crafts and design activities in and out of school once a week in the past 12 months.

	In school (at once a week vs otherwise)			Out of school (at least once a week vs otherwise)		
	OR	95%CI	P-value	OR	95%CI	P-value
***Model 1***						
*Sex*						
Male	REF			REF		
Female	1.31	1.00–1.73	0.052	**2.90**	**2.08–4.03**	**0.000**
*Ethnicity*						
White ethnic	REF			REF		
Ethnic minority	0.91	0.65–1.29	0.603	0.93	0.61–1.42	0.738
*Parents’ marital status*						
Married/in cohabitation	REF			REF		
Single and never married or separated or divorced or widowed	1.04	0.75–1.44	0.819	0.96	0.62–1.47	0.842
*Socio-economic status*						
Higher managerial, administrative and professional occupations	REF			REF		
Intermediate occupations	1.43	0.97–2.09	0.068	1.18	0.72–1.94	0.509
Routine and manual occupations or never worked or long-term unemployed	1.22	0.87–1.72	0.257	1.34	0.85–2.10	0.208
*Parents’ working status*						
Working full-time/part-time	REF			REF		
Not in employment (including students/retired)	0.82	0.55–1.23	0.347	1.06	0.63–1.77	0.837
*Parents’ educational level*						
Degree	REF			REF		
No degree	1.00	0.73–1.35	0.982	0.78	0.51–1.19	0.255
*Levels of area deprivation*						
30% most deprived	0.97	0.69–1.38	0.883	0.93	0.57–1.52	0.784
Medium	REF			REF		
30% least deprived	1.34	0.97–1.86	0.078	1.08	0.70–1.65	0.731
*Tenure*						
Private rented sector or house owning	REF			REF		
Social rented sector	0.97	0.67–1.41	0.871	**1.68**	**1.05–2.70**	**0.032**
*Living area*						
Urban	REF			REF		
Rural	0.97	0.66–1.41	0.861	**1.61**	**1.02–2.54**	**0.041**
***Model 2***						
*+ Parents have done arts and crafts while growing up*	1.26	0.95–1.69	0.111	**1.61**	**1.10–2.35**	**0.014**
*+ Parents have done arts and crafts in the past 12 months*	1.28	0.96–1.69	0.088	**1.97**	**1.36–2.87**	**0.000**
**N**	**1986**			**1986**		

**Table 4:** Logistic regressions estimating the association between socio-demographic backgrounds and children’s archives, museums or heritage sites visits in and out of school 3–4 times a year in the past 12 months.

	In school (at least 3–4 times a year vs otherwise)			Out of school (at least 3–4 times a year vs otherwise)		
	OR	95%CI	P-value	OR	95%CI	P-value
***Model 1***						
*Sex*						
Male	REF			REF		
Female	1.05	0.71–1.57	0.791	**1.45**	**1.11–1.91**	**0.007**
*Ethnicity*						
White ethnic	REF			REF		
Ethnic minority	**1.91**	**1.23–2.94**	**0.004**	**0.52**	**0.35–0.79**	**0.002**
*Parents’ marital status*						
Married/in cohabitation	REF			REF		
Single and never married or separated or divorced or widowed	0.73	0.44–1.23	0.237	1.03	0.74–1.45	0.847
*Parents’ social status*						
Higher managerial, administrative and professional occupations	REF			REF		
Intermediate occupations	1.13	0.65–1.95	0.665	**0.63**	**0.42–0.95**	**0.026**
Routine and manual occupations or never worked or long-term unemployed	0.80	0.48–1.34	0.401	**0.64**	**0.43–0.95**	**0.025**
*Parents’ employment status*						
Working full-time/part-time	REF			REF		
Not in employment (including students/retired)	1.09	0.60–1.96	0.776	0.80	0.49–1.30	0.358
*Parents’ educational level*						
Degree	REF			REF		
No degree	0.90	0.57–1.43	0.662	0.86	0.62–1.20	0.373
*Levels of area deprivation*						
30% most deprived	0.98	0.57–1.68	0.931	0.67	0.44–1.02	0.059
Medium	REF			REF		
30% least deprived	1.34	0.83–2.16	0.228	0.99	0.71–1.40	0.972
*Housing tenure*						
Private rented sector or house owning	REF			REF		
Social rented sector	1.04	0.58–1.85	0.905	**0.58**	**0.37–0.93**	**0.025**
*Living area*						
Urban	REF			REF		
Rural	1.16	0.67–2.00	0.601	1.26	0.85–1.87	0.248
***Model 2***						
*+ Parents have gone to museums or heritage sites while growing up*	1.57	0.94–2.61	0.084	**1.87**	**1.27–2.75**	**0.001**
*+ Parents have gone to museums or heritage sites in the past 12 months*	0.94	0.51–1.75	0.845	**5.59**	**3.03–10.3**	**0.000**
**N**	**1986**			**1986**		

Regarding socio-economic status, compared to those whose parents worked in higher managerial, administrative and professional roles, children whose parents were employed in routine and manual roles or unemployed had a 40% lower odds of engaging in performing arts activities (OR = 0.60, CI = 0.38–0.93). There was also some indication that they had a 36% lower odds of visiting an archive, a museum or a heritage site (OR = 0.64, CI = 0.43–0.95), while children whose parents worked in intermediate roles also had a 37% lower odds of visiting an archive, a museum or a heritage site (OR = 0.63, CI = 0.42–0.95) (Tables 2–4).

Additionally, children who were living in the 30% least deprived areas had a 2 times higher odds of doing performing arts outside school (OR = 2.09, CI = 1.42–3.07). Children who lived in social housing compared with children whose parents rented or owned their houses were 1.7 times higher odds of engaging in arts, crafts and design activities (OR = 1.68, CI = 1.05–2.70) and a 42% lower odds of visiting an archive, a museum or a heritage site (OR = 0.58, CI = 0.37–0.93). Compare with children living in urban areas, those living in rural areas had a 61% higher odds of participating in arts, crafts and design activities outside school (Tables 2–4).

Parental engagement in the related arts was also related to greater children’s engagement. Children had a 68% (OR = 1.68, CI = 1.05–2.70) and 87% (OR = 1.87, CI = 1.27–2.75) higher odds of participating in arts, crafts and design activities and visiting an archive, a museum or a heritage site, respectively, if their parents had done these activities whilst growing up. Children were also more likely to engage in the arts if their parents had done so in the past 12 months, with 2.2 times higher odds for performing arts activities (OR = 2.22, CI = 1.46–3.37), 2 times higher odds for arts, crafts and design activities (OR = 1.97, CI = 1.36–2.87) and 5.6 time higher odds for archive, museum or heritage engagement (OR = 5.59, CI = 3.03–10.3) (Tables 2–4).

However, there was no evidence of differential engagement by parental marital status, whether parents were working or economically inactive, and nor was whether parents had a degree or not (Tables 2–4).

### In-school engagement

In school, there was little evidence of a social gradient across all three activities. Similar to out-of-school engagement, girls had a higher probability of participating in performing arts activities (OR = 1.79, CI = -1.36–2.37). But there were no further differences. However, in contrast to out-of-school engagement, children from an ethnic minority background had a 91% higher odds of visiting an archive, a museum or a heritage site than those from a white ethnic majority background (OR = 1.91, CI = 1.22–2.94) (Tables 2–4).

## Discussion

The main finding of this study is that socio-economic factors predicted arts engagement outside of school, but not in school. Specifically, out of school, children from lower SES families are less likely to participate in the arts (especially performing arts) or engage with culture (e.g. visiting an archive, museum or heritage site) outside of school. Parents’ social status is the most consistent predictor of engagement, but other factors such as living in a more affluent area or living in social housing are also related to participation. Although there has already been evidence showing the social and economic determinants of arts and cultural participation in adults [2, 35–37, 46], this is the first study to examine whether the patterns of engagement are the same for children, and the first to specifically compare differences in engagement at school and outside of school.

There are two main inferences from these findings. First, not only does an individual’s own socio-economic status relate to arts engagement, as previously shown, but our findings suggest that the effects of socio-economic status on participation may transfer into the next generation (as also shown in academic achievement [39] and health outcomes [47]. It is possible that this is because factors such as wealth mean that some families are less able to pay for activities for their children. Indeed, our results for cultural engagement showed that children who live in social housing (which suggests lower levels of wealth) are less likely to go to museums, archives or heritage sites. Whilst some of these activities are free, many require fees either for entry or for travelling to sites. It is also possible, however, that parents of higher SES themselves were more likely to have engaged in the arts as children and were more likely to value arts engagement than parents who do not have past childhood experience of higher levels of engagement. This supports theories and previous studies which suggest that there is an intergenerational transmission of cultural capital from parents to children through tastes and preferences, cultural goods, books or arts [39, 48]. It has been argued that cultural reproduction tends to occur through family upbringing in which the arts are more likely to be recognised by privileged families [39]. In line with this, our results showed that parents’ childhood engagement is a significant predictor of whether children themselves engage outside of school, especially for arts, crafts and design activities and archive, museum or heritage engagement. However, parents’ own current engagement is also a major predictor of children’s engagement outside of school. This may suggest that children who engage in the arts frequently may be more likely to engage with their parents who are likely to encourage and support engagement. Parents of children who are highly engaged in the arts may also actively guide their children to participate in cultural activities.

Another major finding was that there is no evidence of a social gradient in arts participation or cultural engagement in school, nor any evidence that parents’ childhood experiences predict the in-school participation of their children. On the one hand, such a finding might seem obvious as participation at school is often compulsory, rather than voluntary. However, policy developments in the past decade have had a marked effect on provision of the arts in school settings. There has been a steady decrease in arts and cultural participation between 2008 and 2018 among children in the UK [49, 50]. Subjects like Art and Design and Music are not currently included in the national curriculum at secondary Key Stage 4 level (ages around 14–16) [51]. Students in schools where these subjects are not available at this level may have limited opportunities to participate in artistic activities during school lessons where supplies, materials and resources are usually provided or subsidised. Further, more schools have also reduced or completely removed Music in the curriculum as early as for secondary year 7 students (around age 11) or have changed Music as an optional subject at secondary Key Stage 3 level (ages around 11–14) despite Music being in the National Curriculum for these school years [52, 53]. In the absence of obligatory engagement in the arts, there has been an ongoing decrease in voluntary uptake of arts subjects at school [54, 55]. It is encouraging that results still suggest that there are not yet clear social gradients on in-school participation, but it remains to be seen how patterns of inequalities in participation develop in schools as the effects of these policies are realised over the coming years. Not only might the reduction in arts classes pose a challenge to artistic development amongst students that could have implications for other academic-related outcomes such as writing achievement and academic buoyancy [24, 56, 57], but these changes in the delivery of arts opportunities within schools may lead to imbalances in participation amongst young people, widening inequalities in access. Therefore, the findings from this study suggest the importance of school settings for providing equal access to the arts amongst children, in contrast to outside of school, where efforts to enhance participation could risk exacerbating the differences in participation across the social gradient. As a result, policies and practices relating to the promotion of child arts engagement in schools may be a more equal way of trying to ensure universal access to the arts amongst children.

Our research also showed some further noteworthy results. First, while higher SES is positively associated with children performing arts activities and attendance in archives, museums or heritage sites, it is also of note that children from a family with lower levels of wealth (as indicated by living in social housing) were more likely to engage in arts, crafts and design activities out of school. One possibility is that this type of activity is least expensive, not depending as much either on admissions costs and travel, or on tuition. However, future research could consider why we see different socio-economic relationships with certain arts activities but not others and what the barriers or enablers of different types of arts participation amongst children might be. Further, our results show that children living in rural areas are more likely to engage in arts, crafts and design activities outside school than those who lived in urban areas. This is supported by previous studies which show that participation rates in the arts are higher in rural areas than in urban areas [5, 58, 59]. However, no differences are found for other types of engagement. While urban areas may have higher levels of take-up or provision of wide ranging arts and cultural activities (as well as have better accessibility to these activities), rural areas may offer different types of arts and cultural opportunities (e.g. many heritage sites are situated in remote areas). It is also plausible that people living in rural areas may have greater interest for creative arts engagement or have fewer social, personal and financial barriers to engagement than their urban counterparts [59]. However, engagement levels may still vary within cities, towns and villages and across regional locations. Second, female children are likely to engage in the arts, both at schools and outside of school. It is possible this is due to gendered social perception of the arts, as has previously been reported [60–62]. But more research is needed to understand fully why this is and how equal access and engagement can be promoted across genders. Finally, we found that children from an ethnic minority background are more likely to visit archives, museums or heritage sites with school, while less likely to do so outside of school compared to children of white ethnicity. One explanation for this lower out-of-school participation could be related to psychological barriers; it has been suggested that individuals from ethnic minority groups are more likely to have concerns about feeling uncomfortable when engaging in cultural and heritage activities [63]. Alternatively, it is plausible that children from ethnic minority groups may be more likely to be frequently engaged in other cultural activities (e.g. religious or traditional events, cultural community celebrations, parades, festivals), which in turn may lower their attendance in archives, museums and heritage sites. Future research is needed to explore these wider types of cultural behaviours. Also, it is unclear whether the higher engagement in school reflects more opportunities for heritage visits amongst schools with a high proportion of children from ethnic minority backgrounds or a greater uptake amongst these children, perhaps due to increased motivation from parents to consent to such trips due to lower levels of such engagement outside of school. This suggests that in-school arts provision may help alleviate the psychological barrier that might otherwise have occurred when engaging outside of school.

A major strength of this study is that the results were based on a nationally representative sample of children in the UK. Our study also benefited from the rich data collection that includes wide ranging information about respondents’ backgrounds, allowing us to identify socio-demographic factors (including levels of area deprivation) on their arts engagement in school and outside school. Importantly, we controlled for parents’ past and current engagement in the arts, which have been perceived as a strong predictor of children’s engagement. However, we relied on child self-report of arts engagement, which is likely affected by recall bias. Whilst we categorised engagement into frequent vs infrequent engagement rather than relying on the precise recall as to the number of times children had engaged, these results remain to be corroborated by adult self-report in future studies. We only focused on three main types of arts activities that are common amongst children. It may be interesting to examine other types of arts activities such as digital arts and street arts that may have different patterns of engagement. Further, the frequency question in the survey for performing arts categorised activities that are more active (e.g. taking part in a dance performance) with activities that are more receptive (e.g. attending a dance event) together in the same measure. Future study is encouraged to disentangle whether engagement is different across these active and receptive art forms. Relatedly, although the arts categories are based on a previous study on the definition of arts forms for population-based research [2], each specific activity may have different socio-demographic patterns. Additionally, we focused on a particular age range of children. Other studies may like to explore the relationship amongst younger children. We were also unable to identify whether children’s out-of-school activities were engaged with their parents. Understanding who children engage in these activities with could help provide further insights into motivations and opportunities of children’s arts and cultural engagement. Finally, we did not have a measure of what was available for children to engage with in their local area or at school [5]. Future studies could consider what types of schools children attended (some schools in the UK such as academies are not required to follow the national curriculum), as well as whether the distribution of cultural assets or availability of funding for arts activities affects participation rates.

## Conclusions

Overall, this study shows that children’s engagement in the arts in school does not appear to be affected by socio-economic factors, but participation outside of school is graded both socially and depending on parents’ own past experiences as children. School provision of arts and cultural activities therefore appears important in ensuring that access to the arts is equal during crucial developmental periods. Our study suggests that attention to in-school arts provision is necessary, as school creates an equal environment for children to be exposed to various arts activities that is also amenable to interventions to increase participation rates. Given the well-documented evidence on the link between arts engagement and multiple social determinants of health (including child development and educational attainment) and wide-ranging mental and physical health outcomes, ensuring equality of access is an important topic in trying to help reduce social and health inequalities.

## Supporting information

S1 Appendix Activities of children’s arts and cultural participation. (DOCX)

S2 Appendix Activities of parental previous and current engagement in arts and culture. (DOCX)

S1 Table Distribution of children’s engagement in performing arts activities in and out of school by socio-demographic backgrounds. (DOCX)

S2 Table Distribution of children’s engagement in arts, crafts and design activities in and out of school by socio-demographic backgrounds. (DOCX)

S3 Table Distribution of children’s archives, museums or heritage sites visits in and out of school by socio-demographic backgrounds. (DOCX)
