# Supplementary material for: Do socio-demographic factors predict children’s engagement in arts and culture? Comparisons of in-school and out-of-school participation in the Taking Part Survey
Source: PLoS One. 2021 Feb 12;16(2):e0246936. doi: 10.1371/journal.pone.0246936 (PMC7880443; doi:10.1371/journal.pone.0246936)
Supplement: S1 Appendix — (DOCX) [file pone.0246936.s001.docx]

**S1 Appendix. Activities of children’s arts and cultural participation.**

*Performing arts activities*

- Taken part in a dance club
- Taken part in a dance performance
- Created a new dance routine
- Attended a dance event
- Taken part in a dance lesson
- Sang to an audience or rehearsed for a performance
- Practised and rehearsed a musical instrument
- Played a musical instrument to an audience
- Written music (includes writing lyrics or music)
- Attended a live music event
- Taken part in a music lesson
- Rehearsed or performed in a play/drama or drama club
- Taken part in a drama lesson
- Attended theatre performances such as plays, pantomime, opera, musicals or comedy

*Arts, crafts and design activities*

- Painting, drawing, sculpture or model making
- Photography as an artistic activity
- Crafts such as pottery, jewellery making, woodwork, metal work
- Attended exhibition of arts, photography or other craft work
- Taken part in an arts, crafts, design or photography lesson

*Visiting an archive, a museum or a heritage site*

- Visited an archive
- Visited a museum
- Visited a historic building, garden or landscape open to the public
- Visited historical monuments or sites of archaeological interest
- Visited a city or town with historic character
- Visited important modern buildings or public spaces
