# Supplementary material for: Do socio-demographic factors predict children’s engagement in arts and culture? Comparisons of in-school and out-of-school participation in the Taking Part Survey
Source: PLoS One. 2021 Feb 12;16(2):e0246936. doi: 10.1371/journal.pone.0246936 (PMC7880443; doi:10.1371/journal.pone.0246936)
Supplement: S2 Appendix — (DOCX) [file pone.0246936.s002.docx]

**S2 Appendix. Activities of parental previous and current engagement in arts and culture.**

*Parental arts engagement when growing up*

- *Performing arts activities*
- Go to theatre or to see dance or classical music performances
- Play musical instrument(s), act, dance or sing
- *Arts and crafts activities*
- Draw or paint
- *Visiting museums or art galleries or heritage sites*
- Go to museums or art galleries
- Go to historic sites (this includes historic attractions such as old buildings, historic parks and gardens and archaeological sites)

*Parental arts engagement in the past 12 months*

- *Performing arts activities*
- Ballet
- Other dance (not for fitness)
- Written music
- Sang to an audience or rehearse for a performance (not karaoke)
- Played a musical instrument to an audience or rehearse for a performance
- Played a musical instrument for your own pleasure
- Rehearsed or performed in play/drama
- Rehearsed or performed in opera/operetta or musical theatre
- Been to a play/drama
- Been to a pantomime
- Been to a musical
- Been to an opera/operetta
- Been to classical music concert
- Been to a Jazz performance
- Been to other live music event
- Been to ballet
- Been to contemporary dance
- Been to African people’s dance or South Asian and Chinese dance
- Been to other live dance event
- *Arts and crafts activities*
- Painting, drawing, printmaking or sculpture
- Photography as an artistic activity (not family or holiday ‘snaps’)
- Textile crafts such as embroidery, crocheting or knitting
- Wood crafts such as wood turning, carving or furniture making
- Other crafts such as calligraphy, pottery or jewellery making
- Been to an exhibition or collection of art, photography or sculpture
- Been to a craft exhibition (not crafts market)
- *Visiting museums or art galleries or heritage sites*
- Attended a museum or gallery at least once
- Visited a heritage site
