# Supplementary material for: Do socio-demographic factors predict children’s engagement in arts and culture? Comparisons of in-school and out-of-school participation in the Taking Part Survey
Source: PLoS One. 2021 Feb 12;16(2):e0246936. doi: 10.1371/journal.pone.0246936 (PMC7880443; doi:10.1371/journal.pone.0246936)
Supplement: S1 Table — (DOCX) [file pone.0246936.s003.docx]

**S1 Table. Distribution of children’s engagement in performing arts activities in and out of school by socio-demographic backgrounds in % (with weights).**

|  | **In school** | | **Out of school** | |
| --- | --- | --- | --- | --- |
|  | **Less often than once a week** | **At least once a week** | **Less often than once a week** | **At least once a week** |
| *Sex* |  |  |  |  |
| Male | 59.5 | 44.4 | 59.3 | 30.3 |
| Female | 40.5 | 55.6 | 40.7 | 69.7 |
| *Ethnicity* |  |  |  |  |
| Ethnic minority | 27.7 | 26.4 | 29.7 | 20.8 |
| White ethnic | 72.3 | 73.6 | 70.3 | 79.2 |
| *Parental marital status* |  |  |  |  |
| Married/in cohabitation | 68.8 | 68.4 | 66.9 | 72.4 |
| Single and never married or separated or divorced or widowed | 31.2 | 31.6 | 33.1 | 27.6 |
| *Socio-economic status* |  |  |  |  |
| Higher managerial, administrative and professional occupations | 41.9 | 48.4 | 39.4 | 60.1 |
| Intermediate occupations | 21.6 | 23.5 | 23.6 | 20.9 |
| Routine and manual occupations or never worked or long-term unemployed | 36.5 | 28.1 | 37.0 | 19.0 |
| *Parents’ working status* |  |  |  |  |
| Working full-time/part-time | 84.3 | 84.9 | 83.9 | 86.2 |
