# Supplementary material for: Do socio-demographic factors predict children’s engagement in arts and culture? Comparisons of in-school and out-of-school participation in the Taking Part Survey
Source: PLoS One. 2021 Feb 12;16(2):e0246936. doi: 10.1371/journal.pone.0246936 (PMC7880443; doi:10.1371/journal.pone.0246936)
Supplement: S2 Table — (DOCX) [file pone.0246936.s004.docx]

**S2 Table. Distribution of children’s engagement in arts, crafts and design activities in and out of school by socio-demographic backgrounds in % (with weights).**

|  | **In school** | | **Out of school** | |
| --- | --- | --- | --- | --- |
|  | **Less often than once a week** | **At least once a week Total** | **Less often than once a week** | **At least once a week Total** |
| *Sex* |  |  |  |  |
| Male | 54.2 | 47.3 | 55.2 | 30.3 |
| Female | 45.8 | 52.7 | 44.8 | 69.7 |
| *Ethnicity* |  |  |  |  |
| Ethnic minority | 28.3 | 25.8 | 27.1 | 26.0 |
| White ethnic | 71.7 | 74.2 | 72.9 | 74.0 |
| *Parental marital status* |  |  |  |  |
| Married/in cohabitation | 68.8 | 68.4 | 68.8 | 67.8 |
| Single and never married or separated or divorced or widowed | 31.2 | 31.6 | 31.2 | 32.2 |
| *Socio-economic status* |  |  |  |  |
| Higher managerial, administrative and professional occupations | 47.9 | 44.2 | 46.5 | 43.2 |
| Intermediate occupations | 20.2 | 24.8 | 22.6 | 23.7 |
| Routine and manual occupations or never worked or long-term unemployed | 31.9 | 31.0 | 31.0 | 33.1 |
| *Parents’ working status* |  |  |  |  |
| Working full-time/part-time | 83.3 | 85.7 | 84.9 | 83.6 |
| Not in employment (including students/retired) | 16.7 | 14.3 | 15.1 | 16.4 |
| *Parents’ educational level* | |  |  |  |
| Degree | 32.9 | 32.2 | 32.0 | 34.6 |
| No degree | 67.1 | 67.8 | 68.0 | 65.4 |
| *Levels of area deprivation* | |  |  |  |
| 30% most deprived | 32.2 | 29.1 | 30.7 | 29.3 |
| Medium | 38.5 | 35.5 | 36.9 | 36.3 |
| 30% least deprived | 29.4 | 35.5 | 32.4 | 34.3 |
| *Tenure* |  |  |  |  |
| Private rented sector or house owning | 81.8 | 82.9 | 83.6 | 77.3 |
| Social rented sector | 18.2 | 17.1 | 16.4 | 22.7 |
| *Living area* |  |  |  |  |
| Urban | 83.5 | 82.9 | 84.6 | 77.1 |
| Rural | 16.5 | 17.1 | 15.4 | 22.9 |
| *Parents have done arts and crafts while growing up* | | |  |  |
| No | 47.6 | 41.2 | 46.8 | 32.8 |
| Yes | 52.4 | 58.8 | 53.2 | 67.2 |
| *Parents have arts and crafts in the past 12 months* | | |  |  |
| No | 64.0 | 57.2 | 63.5 | 46.5 |
| Yes | 36.0 | 42.8 | 36.5 | 53.5 |
| **Total N** | **921** | **1065** | **1601** | **385** |
