# Supplementary material for: Do socio-demographic factors predict children’s engagement in arts and culture? Comparisons of in-school and out-of-school participation in the Taking Part Survey
Source: PLoS One. 2021 Feb 12;16(2):e0246936. doi: 10.1371/journal.pone.0246936 (PMC7880443; doi:10.1371/journal.pone.0246936)
Supplement: S3 Table — (DOCX) [file pone.0246936.s005.docx]

**S3 Table. Distribution of children’s archives, museums or heritage sites visits in and out of school by socio-demographic backgrounds in % (with weights).**

|  | **In school** | | **Out of school** | |
| --- | --- | --- | --- | --- |
|  | **Less often than 3-4 times a year** | **At least 3-4 times a year Total** | **Less often than 3-4 times a year** | **At least 3-4 times a year Total** |
| *Sex* |  |  |  |  |
| Male | 50.6 | 48.2 | 53.7 | 55.6 |
| Female | 49.4 | 51.8 | 46.3 | 44.4 |
| *Ethnicity* |  |  |  |  |
| Ethnic minority | 25.5 | 36.6 | 32.6 | 17.0 |
| White ethnic | 74.5 | 63.4 | 67.4 | 83.0 |
| *Parental marital status* | |  |  |  |
| Married/in cohabitation | 67.7 | 74.3 | 67.0 | 71.4 |
| Single and never married or separated or divorced or widowed | 32.3 | 25.7 | 33.0 | 28.6 |
| *Socio-economic status* |  |  |  |  |
| Higher managerial, administrative and professional occupations | 45.4 | 49.1 | 39.1 | 57.8 |
| Intermediate occupations | 22.3 | 25.8 | 24.8 | 19.1 |
| Routine and manual occupations or never worked or long-term unemployed | 32.4 | 25.0 | 36.2 | 23.1 |
| *Parents’ working status* |  |  |  |  |
| Working full-time/part-time | 84.8 | 83.6 | 82.9 | 87.7 |
| Not in employment (including students/retired) | 15.2 | 16.4 | 17.1 | 12.3 |
| *Parents’ educational level* | |  |  |  |
| Degree | 31.6 | 38.2 | 29.2 | 38.1 |
| No degree | 68.4 | 61.8 | 70.8 | 61.9 |
| *Levels of area deprivation* | | |  |  |
| 30% most deprived | 30.6 | 29.5 | 36.8 | 19.3 |
| Medium | 37.4 | 32.5 | 34.8 | 40.3 |
| 30% least deprived | 32.0 | 38.0 | 28.4 | 40.4 |
| *Tenure* |  |  |  |  |
| Private rented sector or house owning | 82.1 | 84.3 | 77.9 | 90.3 |
| Social rented sector | 17.9 | 15.7 | 22.1 | 9.66 |
| *Living area* |  |  |  |  |
| Urban | 83.3 | 82.2 | 86.3 | 77.5 |
| Rural | 16.7 | 17.8 | 13.7 | 22.5 |
| *Parents have gone to museums or heritage sites while growing up* | | | |  |
| No | 25.5 | 18.4 | 30.1 | 14.8 |
| Yes | 74.5 | 81.6 | 69.9 | 85.2 |
| *Parents have gone to museums or heritage sites in the past 12 months* | | | |  |
| No | 21.2 | 20.6 | 30.2 | 5.17 |
| Yes | 78.8 | 79.4 | 69.8 | 94.8 |
| **Total N** | **1738** | **248** | **1284** | **702** |
